# Lethal mutagenesis of an RNA plant virus via lethal defection

**DOI:** 10.1038/s41598-018-19829-6

**Published:** 2018-01-23

**Authors:** Luis Díaz-Martínez, Isabel Brichette-Mieg, Axier Pineño-Ramos, Guillermo Domínguez-Huerta, Ana Grande-Pérez

**Affiliations:** 1 Instituto de Hortofruticultura Subtropical y Mediterránea “La Mayora”, Consejo Superior de Investigaciones Científicas-Universidad de Málaga, Área de Genética, Facultad de Ciencias, Campus de Teatinos, 29071 Málaga, Spain; 2 Instituto de Hortofruticultura Subtropical y Mediterránea “La Mayora”, Consejo Superior de Investigaciones Científicas-Universidad de Málaga, Estación Experimental “La Mayora”, 29750 Algarrobo-Costa Málaga, Spain

## Abstract

Lethal mutagenesis is an antiviral therapy that relies on increasing the viral mutation rate with mutagenic nucleoside or base analogues. Currently, the molecular mechanisms that lead to virus extinction through enhanced mutagenesis are not fully understood. Increasing experimental evidence supports the lethal defection model of lethal mutagenesis of RNA viruses, where replication-competent-defectors drive infective virus towards extinction. Here, we address lethal mutagenesis *in vivo* using 5-fluorouracil (5-FU) during the establishment of tobacco mosaic virus (TMV) systemic infections in *N. tabacum*. The results show that 5-FU decreased the infectivity of TMV without affecting its viral load. Analysis of molecular clones spanning two genomic regions showed an increase of the FU-related base transitions A → G and U → C. Although the mutation frequency or the number of mutations per molecule did not increase, the complexity of the mutant spectra and the distribution of the mutations were altered. Overall, our results suggest that 5-FU antiviral effect on TMV is associated with the perturbation of the mutation-selection balance in the genomic region of the RNA-dependent RNA polymerase (RdRp). Our work supports the lethal defection model for lethal mutagenesis *in vivo* in a plant RNA virus and opens the way to study lethal mutagens in plant-virus systems.

## Introduction

The rapid evolution of viruses is a consequence of their compact genomes, high mutation rates, short replicative cycles and large population sizes that together generate highly variable populations termed viral quasispecies^1–3^. Quasispecies properties depend on its viral variants and their positive (complementation) or negative (interference) interaction with each other^4^. The great genotypic and phenotypic varieties of genomes that compose the quasispecies endow it with a high capacity of adaptation to different environments and adverse circumstances and make it possible to extend viral host’s range and to vary the degree of virulence^4–6^. Selection in the mutant spectra of escape mutants resistant to antiviral agents or to the host immune system pressure is still a real challenge in the treatment of viral diseases^7,8^.

A novel antiviral strategy that can circumvent the selection of resistant mutants is lethal mutagenesis, also known as virus entry in error catastrophe, based on the elimination of RNA viruses by increased mutagenesis. Lethal mutagenesis is founded on a theoretical concept, the error threshold, originally introduced by Manfred Eigen and Peter Schuster in their molecular evolution theory that described the population structure and adaptability of primitive replicons^9–11^. According to this theory, there is an upper limit in the error rate during the replication of an organism to maintain the genetic information^10^. Applied to viral infections, violation of the threshold error due to an excess of mutations would lead to the loss of quasispecies structure and elimination of the infection.

Previous studies on quasispecies dynamics showed that the mutant spectrum can influence the behaviour of any variant of the viral quasispecies and suppress it^12^. Mutagenized foot and mouth disease virus (FMDV) pre-extinction RNA interfered and retarded viral infection^13^. According to the suppressor effect of the mutant spectrum observed for lymphocytic choriomeningitis virus (LCMV)^14^, it was discovered in persistent infections in cell culture that the transition to extinction with the mutagenic base analogue 5-fluorouracil (5-FU) was characterized by the gradual decrease of virus-specific infectivity, that is, the infectivity per viral genomic RNA molecule, without a concomitant reduction in the number of genomic RNA molecules of the virus^15–17^. LCMV genomic RNA of the mutagenized population presented greater genetic complexity but a low degree of mutation and the consensus sequence remained invariant^18^. These results led to the model of “lethal defection” for lethal mutagenesis^15^, supported by several experimental^13,16,19–21^ and computational studies^15,16,22,23^. This model contemplates two ways to extinction. In the first, a low mutagenic activity enriches the mutant spectrum with mutated viral genomes, called “defectors”, capable of replication but unable to infect susceptible cells. The defectors, through trans-acting interactions, interfere higher fitness genomes leading to the loss of infectivity and the whole of the quasispecies to extinction. In the second pathway infectivity and replication would be lost simultaneously due to massive mutagenicity without the involvement of defectors.

Some antiviral compounds with mutagenic activity in cell culture appear to have the same activity *in vivo* although the therapeutic mechanism is unknown. One example is ribavirin, a synthetic purine analogue with mutagenic activity^22,24–27^ currently used in combination with interferon for the treatment of hepatitis C virus (HCV). It is unclear whether ribavirin’s mode of action *in vivo* on HCV is mutagenic, immunomodulating or involves other antiviral mechanism^16,28–33^. Increases in the frequency of HCV mutations have been documented^30,34^, although other studies have not associated the antiviral activity of ribavirin with an increased viral error rate^28,32^. Another mutagenic analogue, 5-FU, was able to prevent the *in vivo* establishment of LCMV persistent infection in mouse^35^. After the studies by Loeb *et al*.^36^ on lethal mutagenesis of HIV in cell culture, the analogue 5,6-dihydro-5-aza-20-deoxycytidine (KP-1461) reached the Phase II of a clinical trial in humans. Although KP-1461 altered the mutant spectrum of HIV mutants it did not decrease viral load^37,38^. The combination of two cytosine analogues, gemcitabine (2′-deoxy-2′,2′-difluorocytidine), a ribonucleotide reductase inhibitor, and decitabine (5-aza-2′-deoxycytidine), a mutagenic cytosine analogue similar to KP-1461, decreased HIV infectivity by 73% in cell culture and represents a very promising new approach for HIV extinction^39^. In a mouse model, combination of the two analogues inhibited replication and disease progression caused by another retrovirus, murine leukemia virus (MuLV), although it was not studied whether the antiretroviral activity was associated with increased mutational burden on the virus^40^. A more effective combination therapy with a sequential treatment, first the inhibitor (non-mutagenic) and then the mutagen has been observed for FMDV^22^ and LCMV^27^. Recently, favipiravir (5-fluoro-2-oxo-1H-pyrazine-3-carboxamide)^41,42^ increased the number of mutations in viral RNA and reduced the norovirus load in mice leading in some cases to virus extinction. Further studies are needed *in vivo* to understand the molecular basis of lethal mutagenesis for this antiviral strategy to become a real therapy in the treatment of viral infections.

To date lethal mutagenesis of plant viruses had not been addressed despite being excellent *in vivo* model systems lacking ethical implications and the great complexity, high cost and the requirement of the very specialized labour of *in vivo* animal virus systems. Plants are multicellular eukaryotic organisms and their viruses develop rapid systemic infections, undergo population bottlenecks and have a structure in quasispecies similarly to animal viruses. Therefore, plant virus systems are ideal to tackle the different aspects of the dynamics of quasispecies. *Tobacco mosaic virus* (TMV) belongs to the genus *Tobamovirus* of the family *Virgaviridae*. The genome is unsegmented single stranded RNA (ssRNA) of positive polarity of about 6.4 kb. It contains 4 ORFs encoding a methyltransferase/helicase, an RNA-dependent RNA polymerase (RdRp) (designated 126 K and 183 K, respectively), the MP protein (30 K) and the capsid (CP or 17.5 K). TMV viral RNA has infective capacity^43^ as its genome serves as mRNA for the translation of the RdRp (126 K and 183 K proteins). MP is a non-structural protein that binds to single-stranded nucleic acids, associates with plasmodesmata and it is involved in cell-to-cell and long-distance movement^44^. CP is the only protein of the viral capsid and is responsible for the long-distance movement of the virus within the infected plant^45^.

Here we have studied the molecular basis of lethal mutagenesis of TMV in *N. tabacum* using the base analogue 5-FU. The results show an antiviral effect of 5-FU on TMV related to the mutagenic activity of the analogue. Although the mutation frequency or the number of mutations per molecule did not increase, the complexity of the mutant spectra, the type of changes and the distribution of the mutations were altered. Overall, our results suggest that 5-FU antiviral effect on TMV is associated with the perturbation of the mutation-selection balance in the genomic region of the RdRp. Our work supports the model of lethal defection for *in vivo* lethal mutagenesis in a plant RNA virus.

## Results

### Effect of 5-FU on TMV infectivity and viral load

**Figure 1:**
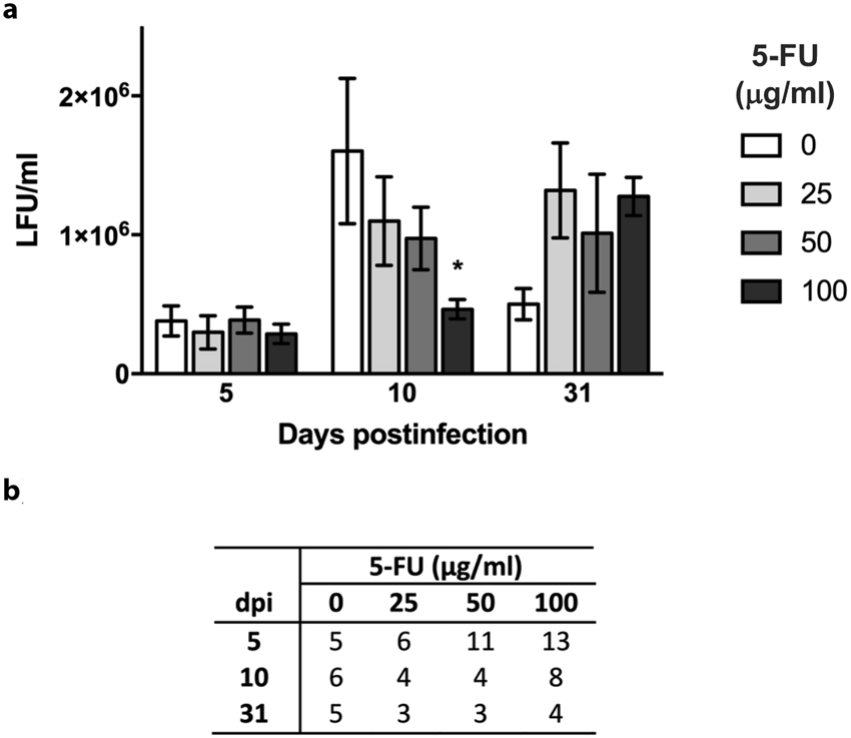
Decrease of TMV infectivity by 5-FU. (**a)** TMV-infected *N. tabacum* cv Samsun nn plants were grown *in vitro* in Magenta® flasks in the absence (0) or the presence of 25, 50 and 100 μg/ml 5-FU. Plants were sampled after 5 and 10 days post inoculation or after a period of 10 days with the analogue and 21 more days in substrate without it (31 dpi). Viral titre (LFU/ml) was determined in saps from whole plants by local lesion assays in *N. tabacum* cv Samsun NN. (**b)** Numbers of assayed plants per 5-FU treatment are indicated. Statistical significance (*) was calculated using the Kruskal-Wallis test for non-parametric distributions. Error bars indicate the standard deviation of biological replicates.

Lethal mutagenesis using base and nucleoside analogues reduces the infectivity of RNA viruses both in cell culture and in animal models^4^. Base analogues such as 2-TU, 5-FU, or the broad spectrum nucleoside analogue ribavirin had been shown to inhibit multiplication of TMV and other plant viruses^46–49^. However, the underlying molecular basis had not been elucidated. To find out if lethal mutagenesis could be responsible for the antiviral activity of 5-FU on TMV, first we analysed TMV infectivity in *N. tabacum* plants subjected to 5-FU treatment. We previously tested the toxicity of the analogue on tobacco plants growing *in vitro* (in Magenta® flasks containing tissue culture medium under sterile conditions) during 10 days in the presence of 25, 50 and 100 μg/ml 5-FU (see Supplementary information and Fig. S1). We found no differences in fresh or dry weight between control and 5-FU treated plants at any dose (one-way Kruskal-Wallis, p < 0.01), ruling out a toxic effect of 5-FU in the plant for the indicated treatments. Then, plants at the four-leaf-stage grown in Magenta® flasks were infected 24 hours after the addition of 25, 50 and 100 μg/ml of 5-FU. Virus infectivity was assayed after 5 and 10 dpi of treatment or after 10 days of treatment followed by 21 days (31 dpi) in the absence of analogue. As shown in Fig. 1 no differences in infectivity among samples were found after 5 days of treatment. However, at 10 dpi increasing 5-FU concentration led to a reduction of TMV infectivity (72% of control) that was significant at the highest dose of 100 µg/ml 5-FU (one-way Kruskal-Wallis, p < 0.01). The observed decrease entails a decay of around 10^6^ infectious particles per ml due to the analogue. However, after removal of 5-FU, viral titres levelled to those of untreated plants (one-way Kruskal-Wallis, p < 0.01). These results suggest a dose-dependent antiviral effect of 5-FU on TMV infectivity after 10 days of treatment.

**Figure 2:**
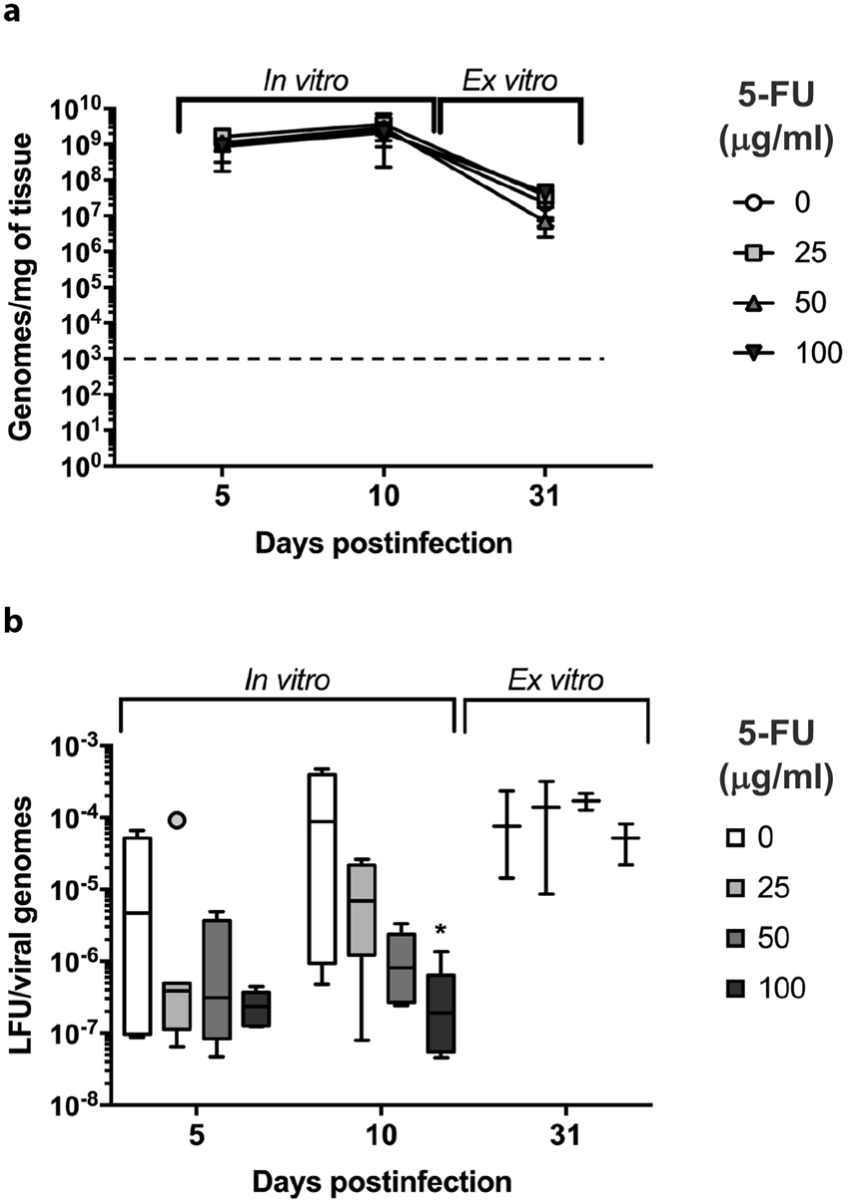
Viral load and specific infectivity of TMV genomic molecules in *N. tabacum* plants treated with 5-FU. (**a)** The number of viral RNA genomic molecules in *N. tabacum* plant extracts of untreated (0) or treated with 25, 50 and 100 μg/ml 5-FU was determined by absolute quantitative RT-PCR. *Ex-vitro* plants were first treated for 10 days *in vitro* in Magenta® flasks with the indicated amounts of 5-FU and then cultured 21 days in substrate without 5-FU. The discontinuous black line parallel to the abscissa indicates the limit of detection of the technique (1.9 × 10^2^ molecules/mg tissue). Error bars indicate the standard deviation of biological replicates. (**b)** Specific infectivity of TMV populations treated with 5-FU. Specific infectivity (infectivity per genomic RNA molecule measured in lesion-forming units, LFU, per genome) of mutant spectra untreated (0) or treated with 25, 50 and 100 μg/ml 5-FU is indicated. Statistically significant differences are indicated by an asterisk (p < 0.01).

The observed antiviral effect of 5-FU could be a result of inhibition of TMV replication. To rule this out, total TMV genomic RNA was quantified in the plant extracts by two-step RTqPCR. Viral loads of approximately 10^9^ genomic molecules per mg of tissue were found in all control and 5-FU-treated samples after 5 and 10 days of treatment (one-way Kruskal-Wallis, p < 0.01) (Fig. 2a). A similar 10-fold decrease in viral load was observed after 10 days of treatment followed by 21 days without drug. These results suggest that TMV replication is not affected by the base analogue and show that the decline in infectivity caused by 5-FU is not due to a reduction in the accumulation of viral genomic molecules.

One of the hallmarks of lethal mutagenesis is the decrease in specific infectivity detected in pre-extinction viral populations^13,18^. As shown in Fig. 2b, TMV populations subjected to a 10-day-treatment with 25, 50 and 100 5-FU µg/ml presented lower specific infectivity than control populations (without 5-FU). The reduction was significant at 100 µg/ml 5-FU (one-way Kruskal-Wallis, p < 0.01). Recovery of specific infectivity was obtained after the removal of the analogue. Note that a similar increment of specific infectivity was observed in control populations during infection suggesting an adaptive process of the stock virus (see Methods for a description of the virus origin). Altogether, our results indicate that the antiviral activity of 5-FU on TMV *in vivo* shows characteristics distinctive of lethal mutagenesis, that is, a decrease of both viral infectivity and specific infectivity while viral load is unaffected.

### 5-FU is mutagenic for TMV *in vivo*

To find out whether the decrease in viral infectivity and specific infectivity was related to a mutagenic activity of the base analogue we looked for mutations in TMV quasispecies. A region of 1320 nucleotides encompassing the 3′ end of 183 K and the whole MP of genomic RNA was amplified by two-step RT-PCR, cloned and 447 clones sequenced. Mutations in clones that were not present in the consensus sequence were analysed. Repeated mutations were considered only once (see Supplementary Table S2 for a complete list of the 190 mutations of control and 5-FU treated quasispecies). Table 1 shows the types of mutations and their percentage after 10 days of treatment with 100 µg/ml 5-FU in each of the two TMV genomic regions. Of a total of 32 mutations 72% were base transitions. Only one deletion and one insertion were found and occurred in the MP region of 5-FU treated samples. Transitions U → C and A → G, i.e. the most common mutations associated to the mutagenic effect of 5-FU, significantly increased both in the RdRp and the MP regions in the presence of 5-FU compared to untreated populations (Two-tailed χ^2^ test, α < 0.05, 3 d.f., RdRp: 29.33, p < 0.0001; MP: 19.12, p < 0.0003).

**Table 1:** Types of mutations found in TMV quasispecies at 10 dpi.

Mutations	0^a^			100^a^		
	RdRp	MP	Total	RdRp	MP	Total
Transitions %						
A → G	0.0	0.0	0.0	33.3 (1)	15.0 (3)	17.4 (4)
G → A	0.0	40.0 (2)	22.2 (2)	0.0	20.0 (4)	17.4 (4)
C → U	25.0 (1)^b^	20.0 (1)	22.2 (2)	0.0	10.0 (2)	8.7 (2)
U → C	50.0 (2)	20.0 (1)	33.3 (3)	66.7 (2)	30.0 (6)	34.8 (8)
Total	75.0 (3)	80.0 (4)	77.8 (7)	100.0 (3)	75.0 (15)	78.3 (18)
Transversions %						
A → C	0.0	0.0	0.0	0.0	0.0	0.0
C → A	0.0	0.0	0.0	0.0	5.0 (1)	4.3 (1)
A → U	0.0	0.0	0.0	0.0	0.0	0.0
U → A	0.0	0.0	0.0	0.0	0.0	0.0
C → G	0.0	0.0	0.0	0.0	0.0	0.0
G → C	0.0	0.0	0.0	0.0	5.0 (1)	4.3 (1)
G → U	0.0	20.0 (1)	11.1 (1)	0.0	0.0	0.0
U → G	25.0 (1)	0.0	11.1 (1)	0.0	5.0 (1)	4.3 (1)
Total	25.0 (1)	20.0 (1)	22.2 (2)	0.0	15.0 (3)	13.0 (3)
Substitutions	100 (4)	100 (5)	100 (9)	100 (3)	90.0 (18)	91.3 (21)
Insertions	0.0	0.0	0.0	0.0	5.0 (1)	4.3 (1)
Deletions	0.0	0.0	0.0	0.0	5.0 (1)	4.3 (1)
Total	100 (4)	100 (5)	100 (9)	100 (3)	100 (20)	100 (23)

^a^Concentration of 5-FU (μg/ml).

^b^Numbers of mutations in brackets.

Further, at 10 dpi the ratio [(A → G) + (U → C)/(G → A) + (C → U)] increased 1.5-fold (25 µg/ml) and 2.24–fold (50 and 100 µg/ml) in the MP region whereas in the RdRp region no (G → A) or (C → U) mutations were detected in samples treated with 50 and 100 µg/ml 5-FU (Supplementary Table S2) and only one (G → A) mutation was found in the 25 µg/ml 5-FU dose. The increase in FU-favoured mutations was not found after 5 days of treatment (Supplementary Table S3). Interestingly, the bias towards (A → G) and (U → C) mutations was maintained in virus populations of plants treated with 5-FU for 10 days followed by a 21-day period without the analogue (Supplementary Table S4).

Regarding the effect of mutations on coding, at 10 dpi with 100 µg/ml 5-FU non-synonymous mutations were in general more abundant than synonymous mutations, especially in the RdRp region (Table 1).

In summary, our results show an increase in transitions associated to the mutagenic action of 5-FU after 10 days of treatment in TMV infected plants.

### Determination of ribonucleotide (NTP) levels in *N. tabacum*

**Figure 3:**
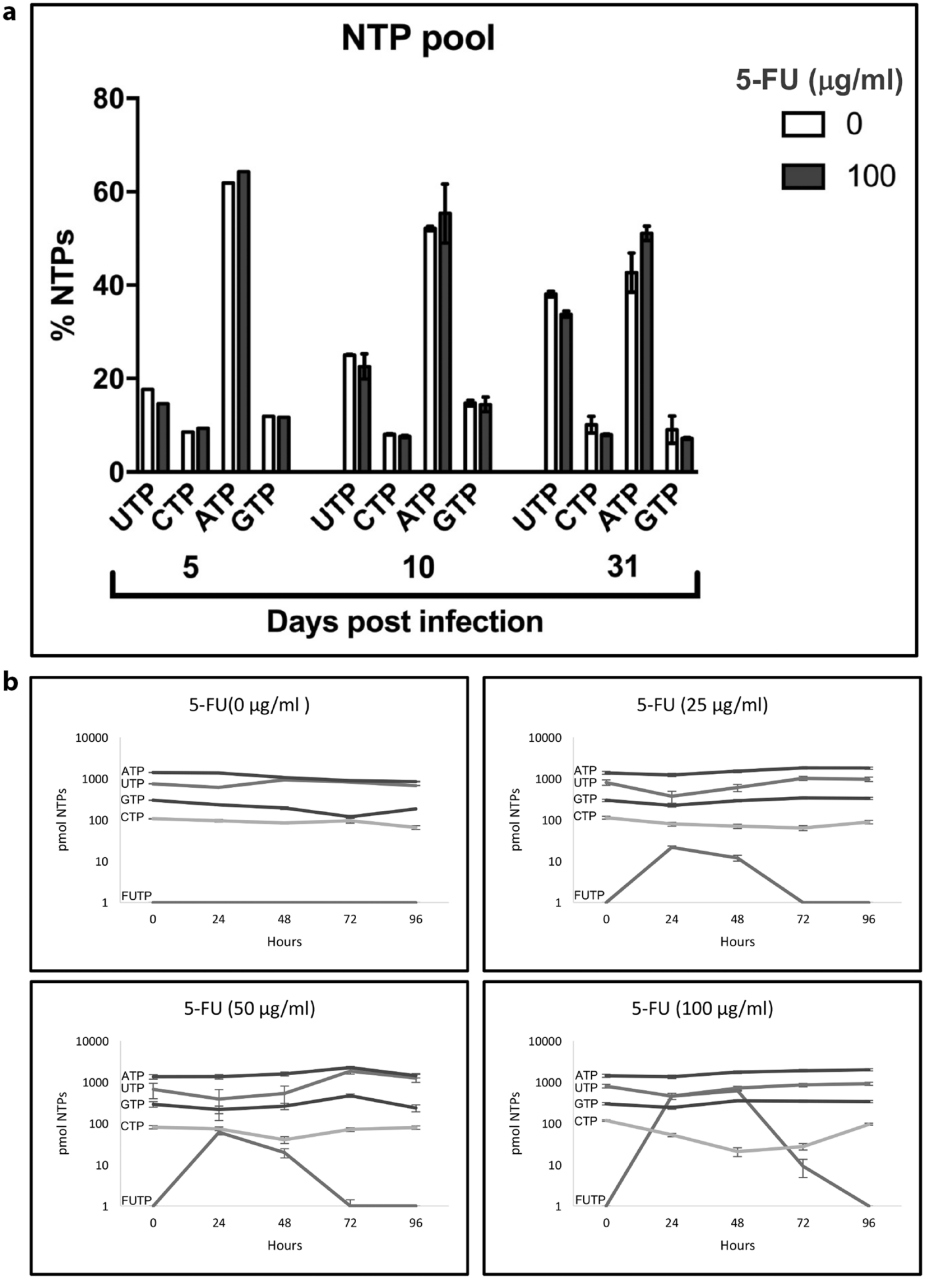
Determination of intracellular ribonucleotide triphosphates (NTP) by HPLC. (**a**) Intracellular extracts of TMV infected plants in the absence or presence of 100 μg/ml 5-FU after 5 and 10 days of treatment *in vitro* in Magenta® flasks and after 10 days of such treatment followed by 21 days in substrate in the absence of 5-FU. Content of CTP, UTP, ATP and GTP is expressed as percentage respect to the total of the four nucleotides. The results correspond to three technical replicates of one biological sample at 5 dpi and three technical replicates of three biological samples at 10 and 31 dpi. The error bars represent the standard deviation of the technical replicates. No significant differences were found at any time for p < 0.01. (**b**) Suspension cultures of non-infected *N. tabacum* BY-2 cells were treated with 0, 25, 50 and 100 µg/ml of 5-FU and the levels of NTP and FUTP after 24, 48, 72 and 96 hours were determined by HPLC.

Imbalance of intracellular nucleotide levels is mutagenic^50^. To ascertain if unbiased levels of intracellular NTP pools *in vivo* could contribute to the mutagenic activity of 5-FU we determined their amounts in *N. tabacum* plants by HPLC. The four NTPs were separated on an anion exchange column and their quantities determined by comparison with their respective standards. Results in Fig. 3a show that no imbalances in the levels of intracellular NTPs were found at any time during or after the treatment with 100 μg/ml 5-FU (one-way Kruskal-Wallis, p < 0.01).

We could not detect the metabolized forms of 5-FU, FUDP or FUTP, even after increasing the initial tissue sample to 4 gr and obtaining amounts of NTPs in the order of nmol. Availability of 5-FU during treatment was confirmed by HPLC analysis of the analogue in the culture medium (results not shown). Further, analysis of intracellular FUTP and NTP levels in suspension cultures of undifferentiated *N. tabacum* BY-2 cells (Fig. 3b) showed that FUTP was detected at 24 hours, reaching sustained levels of 21, 61 and 460 pmol in the presence of 25, 50 and 100 μg/ml 5-FU, respectively, declining after 72–96 h. At the highest dose of analogue, the increase in FUTP was concomitant with the decrease of intracellular CTP (Fig. 3b) whereas ATP, UTP and GTP levels remained constant. The results suggest that mutagenicity of 5-FU on TMV in the plant is not achieved through an imbalance of the NTP pool and that, although the triphosphate form of the analogue is not detectable in the plant, 5-FU can be metabolised into 5-FUTP in *N. tabacum* cells.

### Molecular basis of the decrease in TMV specific infectivity by 5-FU

To understand at the molecular level loss of TMV specific infectivity in the presence of 5-FU, we analysed the genetic complexity and heterogeneity of TMV quasispecies in infected control and 5-FU treated plants. Firstly, the complexity of the mutant spectrum, i.e. the mutational composition of each quasispecies, was analysed by calculating mutation frequency. Mutation frequency of control and 5-FU-treated TMV mutant spectra (Table 2) ranged from 1.65 to 6.28 × 10^−4^ mut/nt. Average error rates of AMV RT^51^ and Pfu DNA Polymerase^52^ have been shown to be 3.3 × 10^−5^ and 1.3 × 10^−6^, respectively, so their contribution to the observed mutation frequencies in our study is small. No significant differences in mutation frequencies were observed at any 5-FU dose (Kruskal-Wallis p < 0.05) or between the two genomic regions (results not shown). Similarly, Shannon entropy, a measure of genetic heterogeneity of the quasispecies, was not significantly different between samples (one-way ANOVA, p < 0.05). Moreover, a mean number of mutations per molecule lower than 1 in all control and 5-FU treated samples (Table 2), indicated a low degree of mutation per genome, characteristic of viral populations undergoing lethal mutagenesis.

**Table 2:** Complexity and heterogeneity of TMV quasispecies in the absence or the presence of 25, 50 and 100 µg/ml 5-FU.

dpi	5-FU (μg/ml)^a^	Clones	Mutations	Nucleotides	Mutation	Shannon	Mutations
				sequenced	frequency^b^	Index	per molecule
5	0 (1)	25	20	31868	6.28 × 10^−4^	0.59	0.80
	0 (2)	45	28	57093	4.90 × 10^−4^	0.53	0.55
	25 (1)	25	11	30331	3.63 × 10^−4^	0.40	0.44
	50 (1)	18	8	21178	3.78 × 10^−4^	0.49	0.44
	100 (1)	20	7	21430	3.27 × 10^−4^	0.32	0.35
	100 (2)	28	6	31464	1.91 × 10^−4^	0.26	0.26
	100 (3)	27	12	29987	4.00 × 10^−4^	0.46	0.44
10	0 (1)	20	9	25600	3.52 × 10^−4^	0.48	0.65
	25 (1)	18	8	22566	3.55 × 10^−4^	0.43	0.44
	50 (1)	21	11	26277	4.19 × 10^−4^	0.48	0.52
	100 (1)	20	14	24951	5.61 × 10^−4^	0.68	0.70
	100 (2)	20	3	24592	1.22 × 10^−4^	0.24	0.20
	100 (3)	19	6	22641	2.65 × 10^−4^	0.38	0.32
31	0 (1)	20	5	24753	2.02 × 10^−4^	0.18	0.25
	0 (2)	22	3	27814	1.08 × 10^−4^	0.26	0.13
	0 (3)	20	4	23783	1.68 × 10^−4^	0.32	0.20
	100 (1)	19	10	24320	4.11 × 10^−4^	0.47	0.53
	100 (2)	20	4	24193	1.65 × 10^−4^	0.18	0.20
	100 (3)	20	11	26694	4.12 × 10^−4^	0.50	0.55
	Total	447	190	547135			

^a^Number of biological replicate in brackets.

^b^Mutations per sequenced nucleotide. Mutations not present in the consensus were counted only once in each mutant spectrum.

The average genetic distance (*d*) of the quasispecies, that is, the mean number of mutations among pairs of randomly selected sequences of the mutant spectrum^53^ was found to be affected by 5-FU. As shown in Table 3, treatment with 100 µg/ml 5-FU for 5 days reduced up to 157-fold the complexity of the MP region. However, the complexity of the RdRp region was not affected. Furthermore, after 10 days the ratio MP/RdRp of *d*_rep_, (*d* calculated after pooling sequences of the replicates) increased from 1.41 (control) to 7.35. The coefficient of variation (CV) of *d*, a measure of the dispersion of *d* between mutant spectra, was calculated separately for each genomic region for control and 5-FU treated samples at any dose. A CV below 1 indicates little variation in the genetic complexity of a set of sequences. In untreated quasispecies CV was close to 1 for RdRp (0.963) and MP (1.330). However, in the presence of 5-FU the RdRp region (0.654) showed lower dispersion of the genetic distances of the sequences compared to the MP region (1.904). These results suggest that there are differences in the genetic complexity of the two genomic regions and that the decrease in specific infectivity maybe linked to these differences. The disparity in *d* between regions was maintained after 21 days in the absence of treatment.

**Table 3:** Average genetic distance (*d*) of the RdRp y MP regions of TMV quasispecies.

dpi	5-FU (μg/ml)^a^	RdRp				MP				MP/RdRp^d^
		*d*	s.e.^b^	*d*_rep_^c^	se^b^	*d*	s.e.^b^	*d*_rep_^c^	s.e.^b^	
**5**	0 (1)	0.00045	0.00026	0.00198	0.00032	0.01709	0.00048	0.06250	0.00647	31.56
	0 (2)	0.00282	0.00047			0.02473	0.00178			
	25 (1)	0.00060	0.00029	0.00060	0.00029	0.05499	0.00364	0.05499	0.00364	91.65
	50 (1)	0.00050	0.00033	0.00050	0.00033	0.04993	0.00349	0.04993	0.00349	99.86
	100 (1)	0.00075	0.00033	0.00060	0.00018	0.00025	0.00013	0.00035	0.00010	0.58
	100 (2)	0.00039	0.00028			0.00026	0.00015			
	100 (3)	0.00070	0.00030			0.00049	0.00843			
**10**	0(1)	0.00075	0.00037	0.00075	0.00037	0.00106	0.00058	0.00106	0.00058	1.41
	25 (1)	0.00021	0.00020	0.00021	0.00020	0.00065	0.00036	0.00065	0.00036	3.09
	50 (1)	0.00038	0.00025	0.00038	0.00025	0.01073	0.00116	0.01073	0.00116	28.2
	100 (1)	0.00000	0.00000	0.00020	0.00010	0.00120	0.00048	0.00147	0.00052	7.35
	100 (2)	0.00060	0.00032			0.00148	0.00048			
	100 (3)	0.00000	0.00000			0.00053	0.00032			
**31**	0 (1)	0.00000	0.00000	0.00022	0.00022	0.00050	0.00048	0.00016	0.00015	0.72
	0 (2)	0.00069	0.00065			0.00000	0.00000			
	0 (3)	0.00024	0.00014			0.00012	0.00012			
	100 (1)	0.00039	0.00026	0.00000	0.00000	0.00111	0.00038	0.00161	0.00081	16.89^e^
	100 (2)	0.00000	0.00000			0.00039	0.00021			
	100 (3)	0.00000	0.00000			0.00659	0.00114			

^a^Number of biological replicate in brackets.

^b^Standard error.

^c^Average genetic distances (d) estimated after pooling all replicates for each treatment and time post infection.

^d^Ratio between *d*_rep_ of MP and RdRp.

^e^Ratio calculated considering only sample 100 (3) (100 μg/ml 5-FU).

A map showing the distribution of mutations after 5 and 10 days of treatment and after 10 days of treatment followed by 21 days in the absence of mutagen in the two genomic regions is depicted in Supplementary Fig. S5. A run test was conducted to evaluate the randomness distribution of mutations in the sequenced area. First, A or U residues, those involved in transitions caused by 5-FU, were found to be distributed randomly in both genomic regions (Supplementary Table S6). In control plants mutations in the MP region were distributed at random whereas in the RdRp region were not (run test, p < 0.05), which suggests a strong restriction of certain positions to mutate. However, in the samples treated with 5-FU the constraints on the RdRp region disappeared (run test, p > 0.05). These results reveal differences in the restrictions of the RdRp and the MP genomic regions to mutate and suggest that TMV in the presence of 5-FU is exploring positions in the RdRp that are not permitted in the absence of mutagen. At 31 dpi, after 21 days in substrate without the pressure of the mutagen (31 dpi) both regions mutations were randomly distributed (Supplementary Table S6).

To find out how selection was acting on the two regions of the viral genome during 5-FU mutagenesis we calculated the rate of non-synonymous substitutions per non-synonymous site (d_ns_) and the rate of synonymous substitutions per synonymous (d_s_) site. The ratio d_ns_/d_s_, shown in Table 4, provides an estimate of the degree and direction of natural selection in untreated and 100 μg/ml 5-FU-treated samples. The results showed again differences between the two regions. Comparing samples at 5 and 10 dpi selection in the control RdRp changed from neutral to negative (purifying selection), whereas in the MP region selection shifted from negative to positive. However, in the presence of 100 μg/ml 5-FU selection both regions went from negative to nearly neutral. These results show that in the absence of analogue natural selection works in opposite directions in the two regions. However, in the presence of 5-FU selection acts in the same way in both regions, that is, at early times of infection purifying selection dominates but later, at 10 dpi, selection becomes neutral. After 21 days in the absence of analogue for the MP region the selection for untreated samples was negative whereas for samples that had been treated for 10 days in the presence of 5-FU selection was strongly positive. Altogether these results suggest that 5-FU *in vivo* alters the mutation-selection equilibrium in both regions disrupting the action of selection.

**Table 4:** Rate of non-synonymous substitutions per non-synonymous site (d_ns_), rate of synonymous substitutions per synonymous (d_s_) site and the ratio d_ns_/d_s_ in RdRp and MP genomic regions of TMV in control and 5-FU-treated samples.

dpi	5-FU	RdRp					MP				
	(μg/ml)	*d*_*ns*_	s.e.^a^	*d*_*s*_	s.e.	*d*_*ns*_ */d*_*s*_	*d*_*ns*_	s.e.	*d*_*s*_	s.e.	*d*_*ns*_/*d*_*s*_
5	0	0.00051	0.00020	0.00047	0.00026	1.08511	0.00108	0.00024	0.00139	0.00046	0.77698
	100	0.00033	0.00015	0.00129	0.00058	0.25581	0.00027	0.00012	0.00047	0.00024	0.57447
10	0	0.00049	0.00034	0.00109	0.00070	0.44954	0.00117	0.00054	0.00076	0.00054	1.53947
	100	0.00021	0.00014	0.00019	0.00019	1.10526	0.00144	0.00094	0.00173	0.00094	0.83237
31	0	0.00031	0.00035	0.00000	0.00000	—	0.00000	0.00000	0.00051	0.00053	0.00000
	100	0.00000	0.00000	0.00000	0.00000	—	0.00216	0.00130	0.00052	0.00029	4.10037

ªStandard Error.

To determine if 5-FU influences the distribution of TMV genetic variability an Analysis of Molecular Variance (AMOVA) test was conducted using Arlequin 3.1. For each genomic region the variation percentage was studied at three levels, variation between 5-FU treatments, between quasispecies and between different sequences in the quasispecies (haplotypes). As shown in Table 5 differences between regions were observed. The genetic variation was 52.8% among treatments for the RdRp region, indicating that the distribution of the genetic variability in this region is strongly influenced by 5-FU. Conversely, in the MP region variation was only found among haplotypes suggesting a random distribution of the genetic variability in this region irrespective of treatments. These results reinforce the idea that the antiviral effect of 5-FU *in vivo* is manifested differently in each of the two genomic regions being the RdRp region more susceptible to mutation. They also suggest that the way TMV explores genetic variability and the space of sequence may be related to the function of the proteins encoded by these regions.

**Table 5:** Analysis of Molecular Variance (AMOVA) of untreated and 5-FU-treated quasispecies at 5 and 10 dpi in the RdRp and MP regions.

Source of variation	TMV Region	d.f.	Sum of squares	Variance components	Percentage of variation	p
Among treatments^a^	RdRp	2	25.783	0.19905	52.85%	<0.05
	MP	1	0.224	−0.00377	<1%	>0.05
Among samples Within treatments^b^	RdRp	10	11.311	0.04453	11.82%	<0.05
	MP	11	6.708	0.01190	3.42%	<0.05
Among sequences Within all samples	RdRp	268	35.652	0.13303	35.32%	<0.05
	MP	292	99.383	0.34035	97.67%	<0.05
Total RdRp Total MP		279 304	72.746 106.315	0.37660 0.34849		

^a^Untreated (0 μg/ml 5-FU) against treated (25, 50 and 100 µg/ml 5-FU) samples (2 groups of treatments).

^b^Untreated (0 μg/ml 5-FU), 25, 50 and 100 µg/ml 5-FU (11 groups of samples).

## Discussion

At present, the molecular mechanisms leading to virus extinction by increased mutagenesis are not fully elucidated. Experimental evidence supports the lethal defection model of RNA viruses. Few studies have addressed lethal mutagenesis *in vivo*. Here, we used a plant RNA virus to understand the molecular basis of loss of infectivity *in vivo*. We have chosen the experimental system of TMV in plants of *N. tabacum*. This system seemed appropriate for several reasons. First, genetic heterogeneity of TMV^54^, and the rate of spontaneous mutation and mutational spectrum of TMV in this host^55^, and the level of diversity of TMV quasispecies in *Nicotiana* from an infectious clone, compatible with a quasispecies structure, had been described^56^. In addition, the effect of bottlenecks on the natural variation of TMV after successive serial passes in the same host^57^ or other hosts^58^ was studied. Furthermore, a decrease in TMV infectivity in *N. tabacum* by the base analogue 5-FU had been documented^59,60^. The treatment was performed one day prior to inoculation of the virus so that the 5-FU main metabolite, FUTP, necessary for 5-FU incorporation into viral RNA, was present in the active phase of virus replication. In analysing the intracellular NTPs content of the plants treated with the analogue for 10 days we did not found imbalances thereof, which discards a mutagenic activity derived from the unavailability of some of the ribonucleotides. Moreover, we were not able to detect the FUTP, even when we increased the amount of starting plant tissue for the extraction until obtaining NTPS quantities on the order of nmole. Failure to detect FUTP could be due either to incapacity of *N. tabacum* cells to metabolize 5-FU or to low availability of FUTP in cells, below the detection level of our method. The first hypothesis was discarded since FUTP was detected in the intracellular fraction of BY-2 cell cultures 24 h after addition of 5-FU, using the same extraction protocol. The second is complicated to determine since we do not know what percentage of 5-FU can reach the leaves and what is the rate of synthesis and degradation of FUTP in them. Spak and colleagues^61^ analysed the amount of phosphorylated forms of the acyclic nucleoside phosphonate tenofovir in *Brassica pekinensis* plants. At 96 h after addition of the tritiated analogue they detected, using a HPLC protocol similar to ours, the maximum concentration of the derivatives equivalent to the NDPs and NTPs. Although the phosphorylated forms represented only 4.5% of the nucleoside added, tenofovir significantly reduced CaMV titre in 3–9 weeks suggesting that the virus is sensitive to very low amounts of the metabolized forms of analogue. It is unknown whether tenofovir mode of action may include mutagenesis. Availability of FUTP in infected cells by increasing the dose of 5-FU or promoting uptake of the mutagen by the plant could lead to higher decreases of virus infectivity leading to overt lethality and virus extinction^62^.

In our experiment, at 5 days of treatment no antiviral activity was detected at any of the doses used, however, at 10 days with 100 μg/ml 5-FU TMV infectivity decreased by 72%. Although the decrease was moderate and significant only for the higher dose of 5-FU, we observed a tendency for a reduction in TMV infectivity with increasing doses of FU. It is likely that a longer treatment, as in the case of the aforementioned study with tenofovir, or with a higher amount of mutagen the reduction of infectivity would have been more remarkable or even reached virus elimination.

Viral load, measured as TMV genomic molecules, was not affected by 5-FU treatment at any dose. However, specific infectivity decreased in the samples with 100 μg/ml 5-FU. These results agree with Barta *et al*.^63^, that in tobacco protoplasts 2-thiouracil (TU) caused a reduction of 10–20% of the specific infectivity of TMV. However, TU did not inhibit ^32^P incorporation into TMV RNA or viral multiplication. Since TU decreased infected cells by 60 to 70%, these authors suggested that TU could express its inhibitory effect without preventing direct inhibition of viral multiplication by generating cell-to-cell defective TMV particles. The decrease in specific infectivity is one of the distinguishing features of the lethal defection model to explain the molecular events underlying virus extinction.

Another hallmark of lethal mutagenesis mediated by defectors is the invariance of the consensus sequence. In our case, we found the mutation G5626A in the consensus sequence of quasispecies treated for 10 days with 100 μg/ml 5-FU (replicate 1). Mutation G5626A was found in 16 of the 20 molecular clones, in some of them associated with other mutations, which indicates high heterogeneity in the mutant spectra. The same mutation appeared in 5 of 20 clones of a control sample at 10 dpi, in this case as a single mutation. Mutation A5627G was only found in the quasispecies treated with 100 μg/ml 5-FU at 31 dpi (replicate 3). These two mutations change the same amino acid localized in MP, the former introduces the replacement Asp242Asn with an acceptability value of 5 (not very drastic) in the SG matrix of Feng *et al*.^64^ and the latter substitution Asp242Gly, with a value of 3. In that region the randomness test found that both at 10 and 31 dpi mutations were randomly distributed, regardless of 5-FU treatment. Further, at 10 dpi in the absence of analogue selection was positive whereas with 100 μg/ml 5-FU the selection became neutral. On the other hand, at 31 dpi the MP is under strong positive selection. We may hypothesise G5626A mutation to be selectively neutral and its imposition, although not total in the consensus sequence, due to genetic drift. Frequent population bottlenecks faced by the virus in this host can decrease its census to units of virions^57^. G5626A mutation is also present in the mutant spectrum of the mentioned control sample at 10 dpi. We cannot rule out that this mutation has been selected in one of the three replicates as a consequence of positive selection by the presence of 5-FU. However, since it lies in the movement protein it is quite unlikely that it is a 5-FU resistance mutation. Given the difficulty of distinguishing between positive and negative selection in a quasispecies^4^, fitness studies of this genotypes in the absence and presence of 5-FU would rule out if resistance to the analogue.

Mutant spectra with reduced infectivity were characterized by no differences in mutation frequency, heterogeneity (Shannon entropy) or the number of mutations per molecule of TMV between 5-FU treated and the controls. However, by analysing in detail the two genomic regions, RdRp and MP, differences in the complexity (estimated as mean genetic diversity) of the mutant spectra were found. Thus, the genetic diversity in the RdRp region was lower than in the MP region in 5-FU treated plants. These differences between regions disappeared after a 21-day regimen without analogue. Lower diversity of RdRp suggests a greater restriction to change in this region. However, there were no differences in the type of mutations between the two regions. The predominant mutations in the mutagenized TMV populations with reduced infectivity were A → G and U → C transitions, as expected due to the action of 5-FU as it replaces uracil residues in RNA. Theoretically all four transitions can be introduced due to the incorporation of 5-FU either to the positive or to the negative strands of RNA viruses. However, for several viral systems there is bias in favour of A → G and U → C transitions^65–68^. This abundance of 5-FU associated mutations did not appear in the samples at 5 dpi, in which there was no significant reduction in viral infectivity. Our results agree with those obtained from Phase IIa of a clinical trial of the analogue KP-1461 (a prodrug of the deoxycytidine analogue KP-1212)^38^ conducted with HIV patients. Reduction of neither viral load nor total number of mutations was detected during the 124-day treatment. However, variations in the mutant spectra of treated individuals were observed, with relative increases of the mutations A → G and G → A, and somewhat less than T → C and C → T, which are those predicted by the mechanism of action of the analogue KP-1212. In that study a sequence of about a thousand nucleotides only from the *gag* region of the HIV genome was analysed so differences in complexity as well as the distribution of mutations between different zones of the genome were not studied.

It should be noted that, unexpectedly, greater complexity of some control quasispecies compared to 5-FU treated quasispecies was observed. In addition, throughout the infection, quasispecies increased their specific infectivity. The fitness gain of a quasispecies in a given environment is the result of the optimization of the mutant spectrum that, through the competition between its components, tends towards a mutation-selection equilibrium. In control populations fitness increase may be due to the clonal origin of the virus and its adaptation to the *in vitro* environment. We observed that the presence of 5-FU altered this balance, especially in the RdRp region normally more restricted to change. RdRp mutant spectrum showed greater exploration in non-permitted positions despite having less complexity than the control. Previous studies of the arenavirus LCMV^65^ remarked the importance of the localization of mutations since the loss of infectivity was not correlated with the increase in mutation frequency. Our results indicate that the RdRp region is more influenced by the presence of 5-FU and substantiates the study of a therapy based on the use of interfering defectors, being the first candidates to be tested *in vivo* the mutations found in the presence of 100 μg/ml 5-FU.

In summary, this is the first study of lethal mutagenesis *in vivo* using a plant RNA virus. The decrease of TMV infectivity due to 5-FU treatment presents characteristics that are the hallmark of the lethal defection: i) a decrease in specific infectivity, ii) no reduction in viral load, iii) changes in the sequence space explored by the mutant spectra and iv) an invariant consensus sequence; and v) low degree of mutation of the TMV genomic molecules. Therefore, our work supports the lethal defection model of lethal mutagenesis *in vivo* and opens the door to this model system to study the kinetics of accumulation of defectors during lethal mutagenesis and the use of viral defector molecules targeting specific regions of the genome. In addition, the TMV- *N. tabacum* system could be used as a non-animal model first approach to study lethal mutagens in monotherapy, in combination or in sequential administration with non-mutagenic antiviral inhibitors.

## Methods

### Plants, cell cultures and viral sources

*Nicotiana tabacum* cv Samsun nn, was used for systemic infections, and *N. tabacum* cv Samsun NN for local lesions assays. When necessary, plants were grown in *ex-vitro*, that is, in pots with a 6:3:1 mix of peat, coconut fiber and litonite, and maintained in growth chamber under long-day conditions (24 °C/16 h day-18 °C/8 h night). Tobacco cv. Samsun nn plants were grown *in vitro* on solid MS medium (4.4 g/l) supplemented with 30 g/l sucrose in Magenta® flasks. Undifferentiated tobacco cv. Bright Yellow-2 (BY-2) (DSMZ PC-1181) cells from callus were cultured in 100 ml Erlenmeyer flasks with MS-BY medium (4.4 g/l MS, 0.2 g/l KH_2_PO_4_, 0,02% 2,4-dichlorophenoxyacetic acid, 10% myo-inositol, and 0,1% HCl-thiamine) under long-day conditions at 22 °C and rotation (100 rpm).

TMV stock was prepared by mechanically inoculating p843pe35TMVr.1 infectious clone (kindly provided by Dr. Edgar Maiß) on a tobacco cv. Samsun NN plant. A single necrotic lesion was used to inoculate a tobacco cv. Samsun nn plant, from which 20 g of symptomatic leaves were homogenised in phosphate buffer (10 mM NaH_2_PO_4_ pH 7.2), and centrifuged at 14,000 × *g* for 15 min at 4 °C. Supernatant was collected and stored at −80 °C.

### 5-Fluorouracil treatment, *in vitro* infection and measurement of toxicity

A stock solution of 10 mg/ml 5-FU was prepared in water and filter-sterilized. Plants grown *in vitro* in Magenta® flasks were transferred at the 3–4 leaf stage to MS containing 5-FU 0, 25, 50 and 100 µg/ml of the analogue in a final volume of 150 ml. One day later, the plants were infected under sterile conditions by rubbing the first true leaf with 5 μl of TMV stock in phosphate buffer containing 10% carburundum, at an MOI of about 50 lesion-forming units (LFU) per plant. After 10 days, plants were transplanted to substrate mixture (what we call *ex-vitro*), without 5-FU. Plants (excluding the root and the inoculated leaf) were sampled at 5, 10, and 31 dpi and stored at −80 °C until further analysis. Sap extracts were prepared by homogenising 100 mg of frozen plant tissue in 100 µl phosphate buffer and centrifuging at 14,000 × *g* for 15 min at 4 °C. Half of the supernatant was used for local lesion assay and half for RNA extraction.

Cultures of non-infected tobacco BY-2 cells at saturation state were treated with the same 5-FU doses to determine NTP and FUTP levels after 24, 48, 72 and 96 hours.

### Extraction of intracellular ribonucleotides and HPLC analysis

Intracellular NTP extraction from plant tissue and BY-2 cells was performed following the method of Pogolotti and Santi (1982) with modifications. Ground fresh tissue of *N. tabacum* (4 g) was homogenized in 4 ml of prechilled 0.6 M trichloroacetic acid (TCA), incubated 10 min on ice and centrifuged at 14800 × g for 10 min at 4 °C. Then, 4 ml of supernatant were filtered in a Strata Phenil (55 μm, 70 A) (Phenomenex) column pre-activated with 3 ml of ethanol and equilibrated with 0.6 M TCA. Four ml of freon:trioctilamine 98% (TOA) 2:1 (v/v) were applied to the column and the eluate was collected in a prechilled tube, vortexed 15 sec and centrifuged at 2500 × g for 2 min at 4 °C. The aqueous phase was transferred to a new tube (pH must be 6.5). Aliquots of 1 ml were frozen in liquid N_2_, lyophilized in a SAVANT SpeedVac, resuspended in 400 μl of MilliQ water, filtered through 0.22 μm Durapore filters (Merck) and stored at −80 °C until analysis by HPLC as described^69^.

One ml of a cell-saturated (approx. 10^6^ cells) BY-2 cell culture was washed twice with PBS at 4 °C and 500 μl of 0.6 M TCA were added. After 10 min incubation on ice, 500 μl of the supernatant were carefully transferred to a new tube and 500 μl of Freon: TOA 4: 1 (v/v) were added. The mixture was stirred vigorously and centrifuged at 14500 × g for 30 sec at 4 °C. Finally, 350 μl of the aqueous phase were collected and stored at −80 °C until analysis by HPLC as described^69^. A standard solution containing 1 nmol of each UTP, CTP, ATP and GTP, was chromatographed prior to the analysis and the amount of each ribonucleotide in samples calculated by Waters Empower^TM^ Chromatography Data Software. Three biological samples and three injections of the same sample were analysed. Nucleotide levels in *N. tabacum* plants extracts were relativized to the total amount of nucleotides of each sample. NAD peak was determined by spectral analysis and used to normalize nucleotide values from BY-2 cells.

### Viral infectivity

TMV infectivity in saps was determined by local lesion assay. Fifty µl of the sap were diluted in a total volume of 800 µl of phosphate buffer and 30 µl of 10-fold dilutions containing 10% (v/v) carborundum were mechanically inoculated on tobacco cv. Samsun NN half-leaves. Six different plants, from five to eight-weeks old, were used to test every four samples. Inoculated plants were maintained under long-day conditions and local lesions were counted after 3–5 dpi.

### Quantification of viral load

Absolute quantitative two-step RT-qPCR was used to measure the number of genomic copies of TMV. For the standard curve, a 474-pb fragment from positions 1367 to 1699 of the TMV genome of p843pe35TMVr.1 was transcribed using T7 RNA polymerase. The reaction was treated with DNase (Ambion) and RNA amount measured by UV spectrophotometry. RT step was performed using 2 µg of total RNA and 20 pmol of reverse primer qTMV1699R (complete list of primers in Supplementary Table S7) at 65 °C for 5 min in a total volume of 7.25 µl. Then, reverse transcriptase 30 U of AMV RT (Promega) and 40 U of RNasin RNA inhibitor (Promega) in a total volume of 20 µl were added to the mix that was incubated for 45 min at 37 °C followed by 5 min at 94 °C. In the second step, 1 µl of the first reaction were mixed with reaction mixture SYBR Green I Master (Roche) and qTMVFor1367 and qTMV1699_R primers (0.5 μM final concentration). Two replicates for each cDNA sample were run using the LightCycler 480 instrument (Roche). For RTqPCR normalization, 25S_universal_F and 25S_universal_R^70^ were used. The PCR protocol consisted of initial denaturalization at 95 °C for 30 s followed by 40 cycles of denaturing at 95 °C for 10 s and primer annealing and extension at 60 °C for 15 s. Standard curves were obtained by linear regression analysis of quantification cycle (C_q_) values of three replicates for dilutions (ranging from 10^9^ to 10^3^ viral copies per μl), with R^2^ of 0.998 and 98% primer efficiency. Number of viral RNA molecules was inferred from this standard curve using Lightcycler software 1.5 (Roche). Additionally, normalization of absolute quantification for each sample was achieved quantifying the 25 S rRNA reference gene^70^.

### RNA extraction, virus amplification, cloning, and sequencing

Total RNA was extracted from ground plant tissue with TRI Reagent® solution (Ambion) following manufacturer’s instructions. cDNA was obtained by two-step RT-PCR performed in a thermocycler Px2 Thermo Electron Corporation, in two steps. The RT reaction (done as indicated in the two-step RTqPCR) used 130 ng of total RNA and 20 pmol of reverse primer A5684R. Five microliters of the first-strand reaction were amplified by touchdown PCR using 1.5 U of *Pfu* DNA polymerase, 10 pmol dNTPs and 20 pmol of A5684R and A4364F primers in a volume of 50 µl. The reaction conditions were denaturation at 95 °C for 2 min, followed by 20 cycles starting at an annealing temperature of 61 °C for 30 s, with a decrease of 0.6 °C per cycle, and 72 °C for 2 min, and 10 additional cycles at an annealing temperature of 48 °C. Amplified DNA fragments were purified with FavorPrepTM GEL/PCR Purification Kit (Favorgen) and A-tailed using BioTaq DNA polymerase (Bioline) and cloned with pGEM®-T Easy Vector System (Promega). Transformed colonies with the correct insert were amplified by rolling-circle amplification (RCA) using Illustra^TM^ TempliPhi kit (GE Healthcare), mixed with 10 μM SP6 or T7 primers and sequenced by Macrogen Inc.

### Sequence analysis and quasispecies characterization

SeqMan software (DNAStar Inc., Madison, WI, USA) was used for sequence assembly and analysis. Mutation frequency was calculated by dividing the number of different substitutions and indels (repeated mutations were computed only once) by the total number of nucleotides sequenced^53^. Genetic distances were estimated for each genomic region by Kimura’s two-parameter method^71^ in MEGA6^72^. Standard error was calculated by the bootstrap method with 1,000 repeats^73^. Normalized Shannon entropy^74^ was calculated with the formula: ${Sn}=-{\sum }_{i}[{(p}_{i}\times {\rm{ln}}{p}_{i})/{\rm{ln}}N]$, whose values range from 0 (all sequences are identical) to 1 (all sequences are different). To address whether positive or negative selection is shaping viral populations, the rates of synonymous substitutions per synonymous site (*d*_*S*_) and of non-synonymous substitutions per non-synonymous site (*d*_*NS*_) site were estimated using the Pamilo-Bianchi-Li method^75^ in MEGA6.

### Statistics

All statistical analysis was performed using IBM SPSS v. 22.0 (SPSS Inc.). The significance of differences in plant dry and fresh weight among treatments mutation frequency, infectivity, viral load, specific infectivity and NTPs imbalances were analysed with non-parametric one-factor Kruskal-Wallis test with p < 0.01. In the case of Shannon entropy ANOVA test with p < 0.05 was used. A standard nonparametric one-way run test with p < 0.05 was conducted to analyse the randomness of the distribution of mutations as a function of genome position. For distribution of genetic variability, an Analysis of Molecular Variance (AMOVA) test was conducted using Arlequin 3.1^76^. A χ^2^ test (α < 0.05) was performed for comparisons of transitions between different genomic regions or treatments.

### Data availability

The datasets generated during and/or analysed during the current study are available from the corresponding author on reasonable request.

## Electronic supplementary material

Supplementary Information

## References

[1] Domingo E, Schuster P (2016). What Is a Quasispecies? Historical Origins and Current Scope. Curr Top Microbiol Immunol.

[2] Domingo E, Schuster P (2016). Quasispecies theory has come of age, and regular updates of the concept of mutation. Introduction. Curr Top Microbiol Immunol.

[3] Nowak MA (1992). What is a quasispecies?. Trends Ecol Evol.

[4] Domingo E, Sheldon J, Perales C (2012). Viral quasispecies evolution. Microbiology and molecular biology reviews: MMBR.

[5] Baranowski E, Ruiz-Jarabo CM, Pariente N, Verdaguer N, Domingo E (2003). Evolution of cell recognition by viruses: a source of biological novelty with medical implications. Adv Virus Res.

[6] Borderia AV (2010). Initial fitness recovery of HIV-1 is associated with quasispecies heterogeneity and can occur without modifications in the consensus sequence. PLoS One.

[7] Perales C, Iranzo J, Manrubia SC, Domingo E (2012). The impact of quasispecies dynamics on the use of therapeutics. Trends in microbiology.

[8] Sanjuan R, Moya A, Elena SF (2004). The distribution of fitness effects caused by single-nucleotide substitutions in an RNA virus. Proc Natl Acad Sci USA.

[9] Eigen M, Schuster P (1977). The hypercycle. A principle of natural self-organization. Part A: Emergence of the hypercycle. Naturwissenschaften.

[10] Eigen M (2002). Error catastrophe and antiviral strategy. Proc Natl Acad Sci USA.

[11] Eigen M (1971). Selforganization of matter and the evolution of biological macromolecules. Naturwissenschaften.

[12] de la Torre JC, Holland JJ (1990). RNA virus quasispecies populations can suppress vastly superior mutant progeny. J Virol.

[13] Gonzalez-Lopez C, Arias A, Pariente N, Gomez-Mariano G, Domingo E (2004). Preextinction viral RNA can interfere with infectivity. J Virol.

[14] Teng MN, Oldstone MB, de la Torre JC (1996). Suppression of lymphocytic choriomeningitis virus–induced growth hormone deficiency syndrome by disease-negative virus variants. Virology.

[15] Grande-Perez A, Lazaro E, Lowenstein P, Domingo E, Manrubia SC (2005). Suppression of viral infectivity through lethal defection. Proceedings of the National Academy of Sciences of the United States of America.

[16] Martin V, Abia D, Domingo E, Grande-Perez A (2010). An interfering activity against lymphocytic choriomeningitis virus replication associated with enhanced mutagenesis. The Journal of general virology.

[17] Arias A (2013). Molecular dissection of a viral quasispecies under mutagenic treatment: positive correlation between fitness loss and mutational load. The Journal of general virology.

[18] Grande-Perez, A., Gomez-Mariano, G., Lowenstein, P. & Domingo, E. Mutagenesis-induced, large fitness variations with an invariant arenavirus consensus genomic nucleotide sequence. *J Virol* **79**, 10451–10459, 10.1128/JVI.79.16.10451-10459.2005 (2005). 10.1128/JVI.79.16.10451-10459.2005 PMC1182645 16051837

[19] Perales C, Mateo R, Mateu MG, Domingo E (2007). Insights into RNA virus mutant spectrum and lethal mutagenesis events: replicative interference and complementation by multiple point mutants. Journal of molecular biology.

[20] Moreno H, Tejero H, de la Torre JC, Domingo E, Martin V (2012). Mutagenesis-mediated virus extinction: virus-dependent effect of viral load on sensitivity to lethal defection. PloS one.

[21] Ojosnegros S (2008). Topology of evolving, mutagenized viral populations: quasispecies expansion, compression, and operation of negative selection. BMC evolutionary biology.

[22] Perales C, Agudo R, Tejero H, Manrubia SC, Domingo E (2009). Potential benefits of sequential inhibitor-mutagen treatments of RNA virus infections. PLoS pathogens.

[23] Iranzo, J. A. & Manrubia, S. C. Stochastic extinction of viral infectivity through the action of defectors. *EPL* **85**, 10.1209/0295-5075/85/18001 (2009).

[24] Brochot E (2007). Effect of ribavirin on the hepatitis C virus (JFH-1) and its correlation with interferon sensitivity. Antivir Ther.

[25] Crotty S (2000). The broad-spectrum antiviral ribonucleoside ribavirin is an RNA virus mutagen. Nat Med.

[26] Maag D, Castro C, Hong Z, Cameron CE (2001). Hepatitis C virus RNA-dependent RNA polymerase (NS5B) as a mediator of the antiviral activity of ribavirin. J Biol Chem.

[27] Moreno H, Grande-Perez A, Domingo E, Martin V (2012). Arenaviruses and lethal mutagenesis. Prospects for new ribavirin-based interventions. Viruses.

[28] Chevaliez S, Brillet R, Lazaro E, Hezode C, Pawlotsky JM (2007). Analysis of ribavirin mutagenicity in human hepatitis C virus infection. J Virol.

[29] Ortega-Prieto AM (2013). Extinction of hepatitis C virus by ribavirin in hepatoma cells involves lethal mutagenesis. PloS one.

[30] Dietz J (2013). Deep sequencing reveals mutagenic effects of ribavirin during monotherapy of hepatitis C virus genotype 1-infected patients. J Virol.

[31] Graci JD, Cameron CE (2006). Mechanisms of action of ribavirin against distinct viruses. Reviews in medical virology.

[32] Lutchman G (2007). Mutation rate of the hepatitis C virus NS5B in patients undergoing treatment with ribavirin monotherapy. Gastroenterology.

[33] Perelson AS, Layden TJ (2007). Ribavirin: is it a mutagen for hepatitis C virus?. Gastroenterology.

[34] Asahina Y (2005). Mutagenic effects of ribavirin and response to interferon/ribavirin combination therapy in chronic hepatitis C. J Hepatol.

[35] Ruiz-Jarabo C (2003). Lethal mutagenesis of the prototypic arenavirus lymphocytic choriomeningitis virus (LCMV). Virology.

[36] Loeb LA (1999). Lethal mutagenesis of HIV with mutagenic nucleoside analogs. Proc Natl Acad Sci USA.

[37] Hicks C (2013). Safety, tolerability, and efficacy of KP-1461 as monotherapy for 124 days in antiretroviral-experienced, HIV type 1-infected subjects. AIDS Res Hum Retroviruses.

[38] Mullins JI (2011). Mutation of HIV-1 genomes in a clinical population treated with the mutagenic nucleoside KP1461. PLoS One.

[39] Clouser CL, Patterson SE, Mansky LM (2010). Exploiting drug repositioning for discovery of a novel HIV combination therapy. J Virol.

[40] Clouser CL (2011). Analysis of the *ex vivo* and *in vivo* antiretroviral activity of gemcitabine. PLoS One.

[41] Arias A, Thorne L, Goodfellow I (2014). Favipiravir elicits antiviral mutagenesis during virus replication *in vivo*. Elife.

[42] Yamada K, Noguchi K, Komeno T, Furuta Y, Nishizono A (2016). Efficacy of Favipiravir (T-705) in Rabies Postexposure Prophylaxis. J Infect Dis.

[43] Gierer A, Schramm G (1956). Infectivity of ribonucleic acid from tobacco mosaic virus. Nature.

[44] Rhee Y, Tzfira T, Chen MH, Waigmann E, Citovsky V (2000). Cell-to-cell movement of tobacco mosaic virus: enigmas and explanations. Molecular plant pathology.

[45] Hilf ME, Dawson WO (1993). The tobamovirus capsid protein functions as a host-specific determinant of long-distance movement. Virology.

[46] Dawson WO, Lozoya-Saldana H (1984). Examination of the mode of action of ribavirin against tobacco mosaic virus. Intervirology.

[47] Lerch B (1987). On the inhibition of plant virus multiplication by ribavirin. Antiviral research.

[48] Dawson WO, Grantham GL (1983). Effect of 2-thiouracil on RNA and protein synthesis in synchronous and asynchronous infections of tobacco mosaic virus. Intervirology.

[49] Holmes FO (1955). Preventive and curative effects of thiouracil treatments in mosaichypersensitive tobacco. Virology.

[50] Mathews CK (2006). DNA precursor metabolism and genomic stability. FASEB journal: official publication of the Federation of American Societies for Experimental Biology.

[51] Roberts JD (1989). Fidelity of two retroviral reverse transcriptases during DNA-dependent DNA synthesis *in vitro*. Molecular and cellular biology.

[52] Cline J, Braman JC, Hogrefe HH (1996). PCR fidelity of pfu DNA polymerase and other thermostable DNA polymerases. Nucleic acids research.

[53] Domingo E (2006). Viruses as quasispecies: biological implications. Current topics in microbiology and immunology.

[54] Rodriguez-Cerezo E, Garcia-Arenal F (1989). Genetic heterogeneity of the RNA genome population of the plant virus U5-TMV. Virology.

[55] Malpica JM (2002). The rate and character of spontaneous mutation in an RNA virus. Genetics.

[56] Schneider WL, Roossinck MJ (2000). Evolutionarily related Sindbis-like plant viruses maintain different levels of population diversity in a common host. J Virol.

[57] Sacristan S, Malpica JM, Fraile A, Garcia-Arenal F (2003). Estimation of population bottlenecks during systemic movement of tobacco mosaic virus in tobacco plants. J Virol.

[58] Schneider WL, Roossinck MJ (2001). Genetic diversity in RNA virus quasispecies is controlled by host-virus interactions. J Virol.

[59] Davern CI, Bonner J (1958). The influence of 5-fluorouracil on tobacco-mosaic virus production in tobacco-leaf discs. Biochim Biophys Acta.

[60] Gleason MK, Fraenkel-Conrat H (1976). Biological consequences of incorporation of 5-fluorocytidine in the RNA of 5-fluorouracil-treated eukaryotic cells. Proc Natl Acad Sci USA.

[61] Spak J (2011). Antiviral activity of tenofovir against Cauliflower mosaic virus and its metabolism in Brassica pekinensis plants. Antiviral research.

[62] Perales C, Domingo E (2016). Antiviral Strategies Based on Lethal Mutagenesis and Error Threshold. Current topics in microbiology and immunology.

[63] Barta A, Sum I, Föglein FJ (1981). 2-Thiouracil does not inhibit TMV replication in tobacco protoplasts. J. Gen. Virol..

[64] Feng DF, Johnson MS, Doolittle RF (1984). Aligning amino acid sequences: comparison of commonly used methods. Journal of molecular evolution.

[65] Grande-Perez A, Sierra S, Castro MG, Domingo E, Lowenstein PR (2002). Molecular indetermination in the transition to error catastrophe: systematic elimination of lymphocytic choriomeningitis virus through mutagenesis does not correlate linearly with large increases in mutant spectrum complexity. Proceedings of the National Academy of Sciences of the United States of America.

[66] Sierra S, Davila M, Lowenstein PR, Domingo E (2000). Response of foot-and-mouth disease virus to increased mutagenesis: influence of viral load and fitness in loss of infectivity. Journal of virology.

[67] Ruiz-Jarabo CM, Ly C, Domingo E, de la Torre JC (2003). Lethal mutagenesis of the prototypic arenavirus lymphocytic choriomeningitis virus (LCMV). Virology.

[68] Agudo R, Arias A, Domingo E (2009). 5-fluorouracil in lethal mutagenesis of foot-and-mouth disease virus. Future medicinal chemistry.

[69] Sanchez-Jimenez C (2012). Mutagen-mediated enhancement of HIV-1 replication in persistently infected cells. Virology.

[70] Mason G, Caciagli P, Accotto GP, Noris E (2008). Real-time PCR for the quantitation of Tomato yellow leaf curl Sardinia virus in tomato plants and in Bemisia tabaci. J Virol Methods.

[71] Kimura M (1980). A simple method for estimating evolutionary rates of base substitutions through comparative studies of nucleotide sequences. J Mol Evol.

[72] Tamura K, Stecher G, Peterson D, Filipski A, Kumar S (2013). MEGA6: Molecular Evolutionary Genetics Analysis version 6.0. Mol Biol Evol.

[73] S, N. M. K. *Molecular Evolution and Phylogenetics*., (Oxford University Press, 2000).

[74] Volkenstein, M. K. *Physical approaches to biological evolution*. (Springer, 1994).

[75] Pamilo P, Bianchi NO (1993). Evolution of the Zfx and Zfy genes: rates and interdependence between the genes. Mol Biol Evol.

[76] Excoffier L, Laval G, Schneider S (2007). Arlequin (version 3.0): an integrated software package for population genetics data analysis. Evol Bioinform Online.

