# Supplementary material for: Lethal mutagenesis of an RNA plant virus via lethal defection
Source: Sci Rep. 2018 Jan 23;8:1444. doi: 10.1038/s41598-018-19829-6 (PMC5780445; doi:10.1038/s41598-018-19829-6)
Supplement: Supplementary file 1 — Supplementary Information [file 41598_2018_19829_MOESM1_ESM.pdf]

# Supplementary information

# Lethal mutagenesis of an RNA plant virus via lethal defection

Luis Díaz-Martínez[1], Isabel Brichette-Mieg[1], Axier Pineño-Ramos[1], Guillermo Domínguez-Huerta[1,2] and Ana Grande-Pérez[1*]

*[1]Instituto de Hortofruticultura Subtropical y Mediterránea "La Mayora", Consejo Superior de Investigaciones Científicas-Universidad de Málaga, Área de Genética, Facultad de Ciencias, Campus de Teatinos, 29071 Málaga, Spain; [2]Instituto de Hortofruticultura Subtropical y Mediterránea "La Mayora", Consejo Superior de Investigaciones Científicas-Universidad de Málaga, Estación Experimental "La Mayora", 29750 Algarrobo-Costa, Málaga, Spain.*

*Corresponding author. Mailing address: Área de Genética, Facultad de Ciencias, Campus de Teatinos, 29071, Málaga, Spain.

Phone: +34 952131677

Fax: +34 952132001

E-mail :agrande@uma.es

**Toxicity of 5-FU on tobacco plants.**

Toxicity of 0, 25, 50 and 100 µg/ml of 5-FU on the tobacco plants grown *in vitro* was determined by measuring dry and fresh weight of tissue (18-23 plants per treatment) after 10 days of treatment and compared to the control. No differences in fresh or dry weight were observed between control and 5-FU treated plants at any dose (one-way ANOVA, $p<0.05$), ruling out a *in vivo* toxic effect of 5-FU for the indicated treatments.

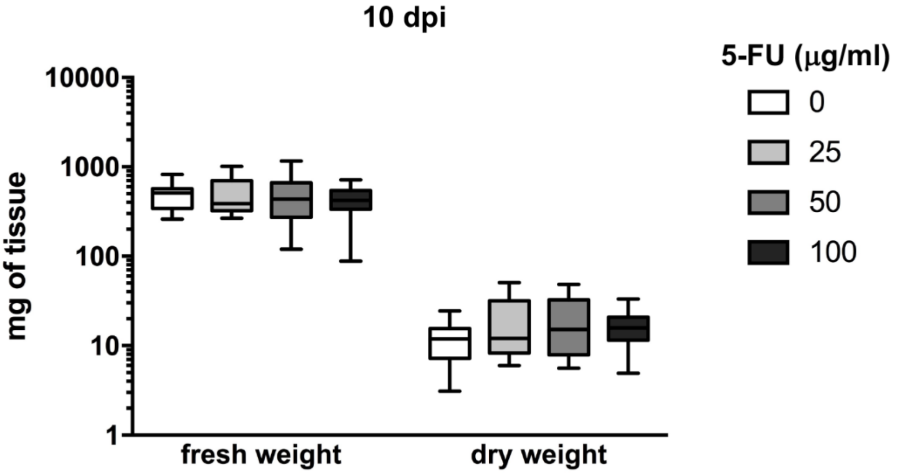


**Figure S1. Toxicity of 5-FU for *N. tabacum* plants.** Plants grown *in vitro* were treated at the four-leaf stage with 0, 25, 50 and 100 µg/ml 5-FU for 10 days. The fresh and dry weight was determined per mg of plant tissue. For each treatment 18 to 23 plants were analysed.

**Table S2. List of all mutations and amino acid changes**

| 5 dpi | Mutation | Codon change | Amino acid change | Region |
|---|---|---|---|---|
| 0 (1) | U4835C | GGU→GGC | Gly1589Gly | RdRp |
| | U4844C | GUU→GUC | Val1592Val | RdRp |
| | A4887U | AGA→UGA | Arg1607STOP[a] | RdRp |
| | A4925G | AAA→AAG | Lys8Lys | MP |
| | U4942A | GUU→GAU | Val14Asp | MP |
| | A4956Del | AAA→AAA | Lys18Frameshift | MP |
| | G4963A | GGA→GAA | Gly21Glu | MP |
| | U5051C | AUU→AUC | Ile50Ile | MP |
| | U5104C | CUG→CCG | Cys68Pro | MP |
| | C5126U | CAC→CAU | His75His | MP |
| | C5143U | GCC→GUC | Ala81Val | MP |
| | A5247G | AAG→GAG | Lys115Glu | MP |
| | C5259U | CAA→UAA | Gln119STOP[a] | MP |
| | U5398C | UUA→UCA | Leu166Ser | MP |
| | A5427G | AGA→GGA | Arg175Gly | MP |
| | A5446G | GAG→GGG | Glu182Gly | MP |
| | A5449G | AGA→GGA | Arg183Gly | MP |
| | A5528G | AAA→AAG | Lys209Lys | MP |
| | U5536A | AUC→AAC | Ile212Asn | MP |
| | G5635C | AGG→ACG | Arg244Thr | MP |

| | | | | |
|---|---|---|---|---|
| 0 (2) | U4441C | AUU→ACU | Ile1456Thr | RdRp |
| | C4492U | GCC→GUC | Ala1473Val | RdRp |
| | C4515U | CUG→UUG | Leu1481Leu | RdRp |
| | C4542U | CCG→UCG | Pro1490Ser | RdRp |
| | A4770G | AAU→GAU | Asn1566Asp | RdRp |
| | C4788U | CAG→UAG | Gln1572STOP[a] | RdRp |
| | A4789U | CAG→CUG | Gln1572Leu | RdRp |
| | A4848G | AAA→GAA | Lys1592Glu | RdRp |
| | U4882Del | CUU→CUU | Leu1603Frameshift | RdRp |
| | U4884C | UUU→CUU | Phe1604Leu | RdRp |
| | A4923G | GGA→GGG | Gly4Gly | MP |
| | C5048U | UCA→UUA | Ser46Leu | MP |
| | G5166U | GUG→GUU | Val85Val | MP |
| | U5264C | GUC→GCC | Val118Ala | MP |
| | U5300C | AUG→ACG | Met130Thr | MP |
| | U5321Del | UUA→UAG | Leu137STOP[a] | MP |
| | U5379C | UUU→UUC | Phe156Phe | MP |
| | U5393C | AUU→ACU | Ile161Thr | MP |
| | A5401U | AGA→UGA | Arg164STOP[a] | MP |
| | U5483A | GUU→GAU | Val191Asp | MP |
| | U5508C | CCU→CCC | Pro199Pro | MP |

| 5 dpi | Mutation | Codon change | Amino acid change | Region |
|---|---|---|---|---|
| | G5571Del | GGG→GGA | Gly220Frameshift | MP |
| | A5575Del | AUU→UUA | Ile222Frameshift | MP |
| | G5601A | CCG→CCA | Pro230Pro | MP |
| | A5656G | AAU→GAU | Asn249Asp | MP |
| | A5657Del | AAU→AUU | Asn249Frameshift | MP |
| | A5662G | AUC→GUC | Ile251Val | MP |
| | C5664A | AUC→AUA | Ile251Ile | MP |
| | C5664U | AUC→AUU | Ile251Ile | MP |

| | | | | |
|---|---|---|---|---|
| 25 (1) | G4530U | GGU→UGU | Gly1488Cys | RdRp |
| | C4542A | CCG→ACG | Pro1492Thr | RdRp |
| | A4588U | AAA→AUA | Lys1507Ile | RdRp |
| | U4865C | UAU→UAC | Tyr1599Tyr | RdRp |
| | A5029G | AAU→AGU | Asn43Ser | MP |
| | G5161U | GGU→UGU | Gly87Cys | MP |
| | G5162Del | GGU→GUG | Gly87Frameshift | MP |
| | U5163C | GGU→GGC | Gly87Gly | MP |
| | A5243U | AAG→AUG | Lys114Met | MP |
| | U5257G | UUC→UGC | Phe119Cys | MP |
| | A5662G | AUC→GUC | Ile254Val | MP |

| | | | | |
|---|---|---|---|---|
| 50 (1) | C4385U | AUC→AUU | Ile1439Ile | RdRp |
| | A4596Ins | AAA→AAA | Lys1510Frameshift | RdRp |
| | G4812A | GUU→AUU | Val1582Ile | RdRp |
| | G4820U | AAG→AAU | Lys1584Asn | RdRp |
| | A5306Del | AAC→AAG | Asn135Frameshift | MP |
| | U5561C | GUC→GCC | Val220Ala | MP |
| | A5661G | UUA→UUG | Leu253Leu | MP |
| | C5664A | AUC→AUA | Ile254Ile | MP |

| | | | | |
|---|---|---|---|---|
| 100 (1) | G4501U | GGU→GUU | Gly1476Val | RdRp |
| | A4555C | CAC→CCC | His1494Pro | RdRp |
| | C4671U | CUA→UUA | Leu1533Leu | RdRp |
| | U4866C | UUG→CUG | Leu1600Leu | RdRp |
| | U5359G | UGU→GGU | Cys153Gly | MP |
| | G5360A | UGU→UAU | Cys153Tyr | MP |
| | C5664U | AUC→AUU | Ile254Ile | MP |

| | | | | |
|---|---|---|---|---|
| 100 (2) | U4494G | UUU→GUU | Phe1476Val | RdRp |
| | G4676A | AAG→AAA | Lys1536Lys | RdRp |
| | G5338A | GUG→AUG | Val146Met | MP |
| | G5338U | GUG→UUG | Val146Leu | MP |
| | U5530C | UUU→CUU | Phe210Leu | MP |
| | C5664U | AUC→AUU | Ile254Ile | MP |

| 5 dpi | Mutation | Codon change | Amino acid change | Region |
|---|---|---|---|---|
| 100 (3) | A4428G | AAC→GAC | Asn1454Asp | RdRp |
| | C4456U | GCC→GUC | Ala1463Val | RdRp |
| | G4544U | CCG→CCU | Pro1492Pro | RdRp |
| | A4576U | AAU→AUU | Asn1503Ile | RdRp |
| | U4754C | GUU→GUC | Val1562Val | RdRp |
| | A4960Del | AUG→UGG | Met20Frameshift | MP |
| | U5019C | GUU→GUC | Val39Val | MP |
| | C5148U | GAC→GAU | Asp82Asp | MP |
| | U5406C | AAU→AAC | Asn186Asn | MP |
| | A5581U | AGU→GGU | Ser227Gly | MP |
| | C5599U | CCG→UCG | Pro233Ser | MP |
| | A5662U | AUC→UUC | Ile254Phe | MP |

| 10 dpi | Mutation | Codon change | Amino acid change | Region |
|---|---|---|---|---|
| 0 (1) | U4577C | AAU→AAC | Asn1503Asn | RdRp |
| | U4593G | UUU→GUU | Phe1509Val | RdRp |
| | U4748C | UGU→UGC | Cys1560Cys | RdRp |
| | C4881U | CUU→UUU | Leu1605Phe | RdRp |
| | U5022C | GAU→GAC | Asp40Asp | MP |
| | C5100U | UAC→UAU | Tyr66Tyr | MP |
| | G5122A | GUC→AUC | Val74Ile | MP |
| | G5162U | GGU→GUU | Gly87Val | MP |
| | G5626A | GAU→AAU | Asp242Asn | MP |
| 25 (1) | G4710A | GAU→AAU | Asp1548Asn | RdRp |
| | A4960Del | AUG→UGG | Met20Frameshift | MP |
| | U5053C | UCA→CCA | Ser51Pro | MP |
| | G5122A | GUC→AUC | Val74Ile | MP |
| | G5313A | UGG→UGA | Trp137STOP[a] | MP |
| | C5599U | CCG→UCG | Pro233Ser | MP |
| | U5613C | UAU→UAC | Tyr237Tyr | MP |
| | A5656G | AAU→GAU | Asn252Asp | MP |
| 50 (1) | A4589G | AAA→AAG | Lys1507Lys | RdRp |
| | G4888U | AGA→AUA | Arg1607Ile | RdRp |
| | U5355C | GGU→GGC | Gly151Gly | MP |
| | U5037C | CAU→CAC | His45His | MP |
| | G5200C | GCC→CCC | Ala100Pro | MP |
| | U5251C | UUU→CUU | Phe117Leu | MP |
| | G5293U | GAC→UAC | Asp131Tyr | MP |
| | C5365T | CUU→UUU | Leu155Phe | MP |
| | A5552Del | AAG→AGA | Lys217Frameshift | MP |

| 10 dpi | Mutation | Codon change | Amino acid change | Region |
|---|---|---|---|---|
| | C5664A | AUC→AUA | Ile254Ile | MP |
| | C5664U | AUC→AUU | Ile254Ile | MP |
| 100 (1) | A4528G | AAG→AGG | Lys1487Arg | RdRp |
| | U4883C | CUU→CUC | Leu1605Leu | RdRp |
| | U4896C | UUU→CUU | Phe1610Leu | RdRp |
| | U4974Ins | UUA→UUU | Leu24Frameshift | MP |
| | G4983C | AUG→AUC | Met27Ile | MP |
| | A5109G | UUA→UUG | Leu69Leu | MP |
| | A5132Del | GAG→GGU | Glu77Frameshift | MP |
| | G5179A | GUG→AUG | Val93Met | MP |
| | A5207G | GAG→GGG | Glu102Gly | MP |
| | C5562A | GUC→GUA | Val220Val | MP |
| | G5626A | GAU→AAU | Asp242Asn | MP |
| | G5633A | GGA→GAA | Gly244Glu | MP |
| | G5635A | GGA→AGA | Gly245Arg | MP |
| | U5644C | UUU→CUU | Phe248Leu | MP |
| 100 (2) | U5037C | CAU→CAC | His45His | MP |
| | U5223C | UCU→UCC | Ser107Ser | MP |
| | U5502C | GAU→GAC | Asp200Asp | MP |
| 100 (3) | U5444G | GUG→GGG | Val181Gly | MP |
| | C5562U | GUC→GUU | Val220Val | MP |
| | U5593C | UUA→CUA | Leu231Leu | MP |
| | U5631C | UUU→UUC | Phe243Phe | MP |
| | A5662G | AUC→GUC | Ile254Val | MP |
| | C5664U | AUC→AUU | Ile254Ile | MP |

| 31 dpi | Mutation | Codon change | Amino acid change | Region |
|---|---|---|---|---|
| 0 (1) | U4442C | AUU→AUC | Ile1458Ile | RdRp |
| | U4502C | GGU→GGC | Gly1478Frameshift | RdRp |
| | A4956Del | ACA→ACA | Thr18Thr | MP |
| | U5022C | GAU→GAC | Asp40Asp | MP |
| | C5295G | GAC→GAG | Asp131Glu | MP |
| 0 (2) | G4463A | AUG→AUA | Met1465Ile | RdRp |
| | G4550A | GUG→GUA | Val1494Val | RdRp |
| | C4652U | UGC→UGU | Cys1528Cys | RdRp |
| | A4783C | UAC→UCC | Tyr1572Ser | RdRp |
| | A4537G | GAG→GGG | Glu1490Gly | RdRp |

| 31 dpi | Mutation | Codon change | Amino acid change | Region |
|---|---|---|---|---|
| 0 (3) | U4615C | UUU→UCU | Phe1516Ser | RdRp |
| | C5148G | GAC→GAG | Ser82Glu | MP |
| | | | | |
| 100 (1) | U4386C | UGG→CGG | Trp1440Arg | RdRp |
| | A4519G | UAC→UGC | Tyr1484Cys | RdRp |
| | C4975Del | CCG→CGU | Pro25Frameshift | MP |
| | A5045G | GAG→GGG | Glu48Gly | MP |
| | U5094C | AGU→AGC | Ser64Ser | MP |
| | U5280C | GCU→GCC | Ala126Ala | MP |
| | G5333U | AGA→AUA | Arg144Ile | MP |
| | A5440G | AAC→GAC | Asn180Asp | MP |
| | U5644C | UUU→CUU | Phe248Leu | MP |
| | A5661C | UUA→UUC | Leu253Phe | MP |
| | | | | |
| 100 (2) | U5516C | AUC→ACC | Ile205Thr | MP |
| | U5619C | AAU→AAC | Asn239Asn | MP |
| | U5663G | AUC→AGC | Ile254Ser | MP |
| | C5664A | AUC→AUA | Ile254Ile | MP |
| | | | | |
| 100 (3) | U4389C | UAU→CAU | Tyr1441His | RdRp |
| | U4992A | CCU→CCA | Pro30Pro | MP |
| | U5115C | GGU→GGC | Gly71Gly | MP |
| | C5124U | GUC→GUU | Val74Val | MP |
| | U5337A | AAU→AAA | Asn145Lys | MP |
| | U5337A | AAU→AAA | Asn145Lys | MP |
| | U5361C | UGU→UGC | Cys153Cys | MP |
| | U5381C | GUG→GCG | Val60Ala | MP |
| | U547C | GAU→GAC | Asp195Asp | MP |
| | A5627G | GAU→GGU | Asp242Gly | MP |
| | G5633U | GGA→GUA | Gly244Val | MP |

[a] Truncated protein.

**Table S3. Types of mutations found in TMV quasispecies at 5 dpi.**

| Mutations at 5 dpi | 0[a] | | | 100[a] | | |
|---|---|---|---|---|---|---|
| | RdRp | MP | Total | RdRp | MP | Total |
| **Transitions %** | | | | | | |
| A→G | 15.4 | 25.0 | 22.4 | 9.1 | 7.1 | 8.0 |
| G→A | 0.0 | 5.6 | 4.1 | 9.1 | 14.3 | 12.0 |
| C→U | 30.8 | 13.9 | 18.4 | 18.2 | 28.6 | 24.0 |
| U→C | 30.8 | 22.2 | 24.5 | 18.2 | 28.6 | 24.0 |
| Total | 76.9 | 66.7 | 69.4 | 54.5 | 78.6 | 68.0 |
| **Transversions %** | | | | | | |
| A→C | 0.0 | 0.0 | 0.0 | 9.1 | 0.0 | 4.0 |
| C→A | 0.0 | 2.8 | 2.0 | 0.0 | 0.0 | 0.0 |
| A→U | 15.4 | 2.8 | 6.1 | 9.1 | 7.1 | 8.0 |
| U→A | 0.0 | 8.3 | 6.1 | 0.0 | 0.0 | 0.0 |
| C→G | 0.0 | 0.0 | 0.0 | 0.0 | 0.0 | 0.0 |
| G→C | 0.0 | 2.8 | 2.0 | 0.0 | 0.0 | 0.0 |
| G→U | 0.0 | 2.8 | 2.0 | 18.2 | 7.1 | 12.0 |
| U→G | 0.0 | 0.0 | 0.0 | 9.1 | 0.0 | 4.0 |
| Total | 15.4 | 19.4 | 18.4 | 45.5 | 14.3 | 28.0 |
| | | | | | | |
| **Substitutions** | 92.3 | 86.1 | 87.8 | 100 | 92.9 | 96.0 |
| **Insertions** | 0.0 | 0.0 | 0.0 | 0.0 | 0.0 | 0.0 |
| **Deletions** | 7.7 | 13.9 | 12.2 | 0.0 | 7.1 | 4.0 |
| **Total** | 100 | 100 | 100 | 100 | 100 | 100 |

[a] Concentration of 5-FU (µg/ml).

**Table S4. Types of mutations found in TMV quasispecies at 31 dpi.**

| Mutations at 31 dpi | 0[a] | | | 100[a] | | |
|---|---|---|---|---|---|---|
| | RdRp | MP | Total | RdRp | MP | Total |
| **Transitions %** | | | | | | |
| A→G | 12.5 | 0 | 8.3 | 33.3 | 14.2 | 16.6 |
| G→A | 25.0 | 0 | 16.6 | 0 | 0 | 0 |
| C→U | 12.5 | 0 | 8.3 | 0 | 4.7 | 4.1 |
| U→C | 37.5 | 25 | 33.3 | 66.6 | 38.1 | 41.6 |
| Total | 87.5 | 25 | 66.6 | 100 | 57.1 | 62.5 |
| **Transversions %** | | | | | | |
| A→C | 12.5 | 0 | 8.3 | 0 | 4.7 | 4.1 |
| C→A | 0 | 0 | 0 | 0 | 4.7 | 4.1 |
| A→U | 0 | 0 | 0 | 0 | 0 | 0 |
| U→A | 0 | 0 | 0 | 0 | 14.2 | 12.5 |
| C→G | 0 | 50 | 16.6 | 0 | 0 | 0 |
| G→C | 0 | 0 | 0 | 0 | 0 | 0 |
| G→U | 0 | 0 | 0 | 0 | 9.5 | 8.3 |
| U→G | 0 | 0 | 0 | 0 | 4.7 | 4.1 |
| Total | 12.5 | 50 | 25 | 0 | 38.0 | 33.3 |
| | | | | | | |
| Substitutions | 100 | 75 | 91.6 | 100 | 95.2 | 95.8 |
| Insertions | 0 | 0 | 0 | 0 | 0 | 0 |
| Deletions | 0 | 25 | 8.3 | 0 | 4.7 | 4.1 |
| Total | 100 | 100 | 100 | 100 | 100 | 100 |

[a] Concentration of 5-FU (µg/ml).

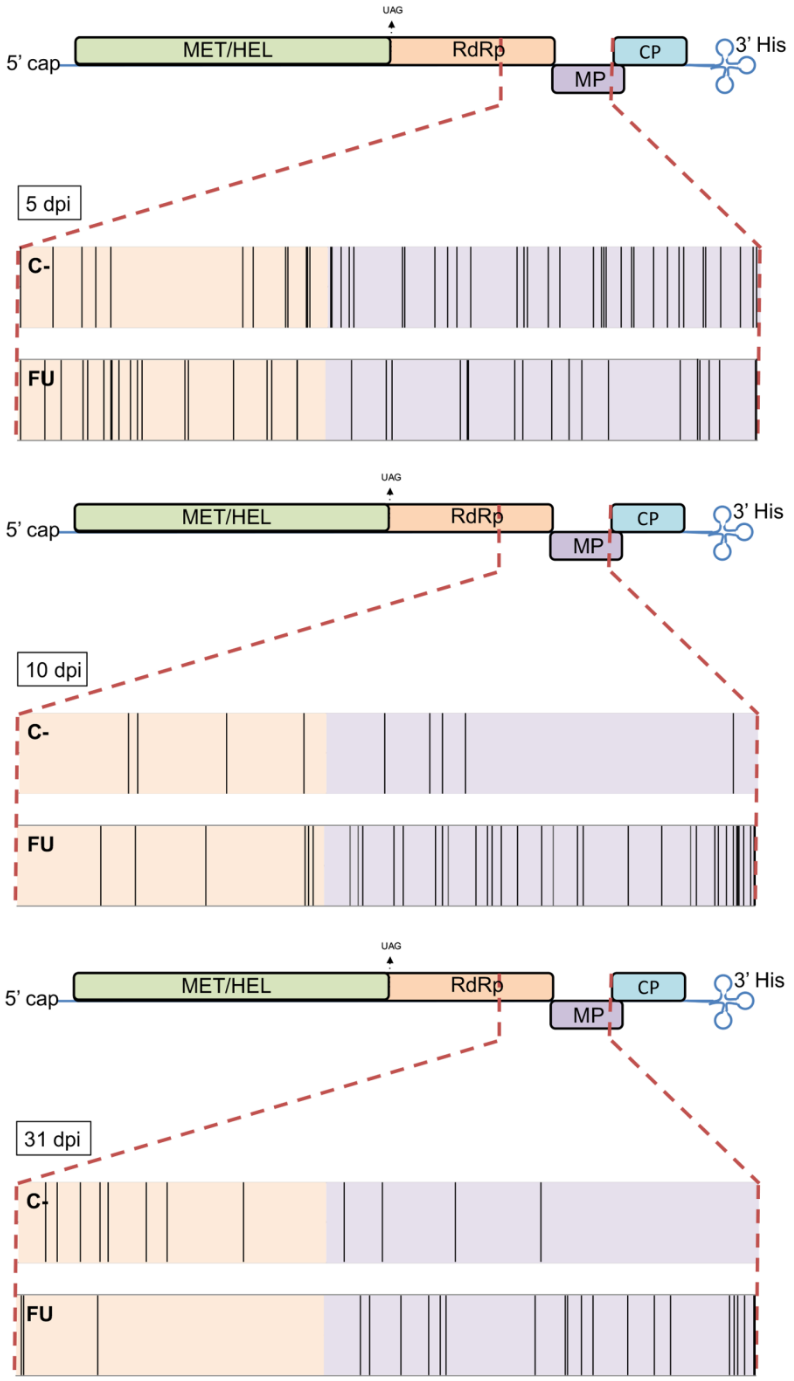


**Figure S5. Map showing the localization of the point mutations present in the RdRp and MP regions sequenced in TMV quasispecies untreated (C-) and treated with 25, 50, and 100 µg/ml 5-FU (FU) after 5 (5dpi) and 10 days of treatment (10 dpi), or after a 10 day-treatment with the indicated amounts of 5-FU followed by 21 days without 5-FU (31 dpi).** At the top of each panel the TMV RNA genome is shown indicating the cap at the 5' end, its four ORFs, namely, methyltransferase/helicase (MET/HEL), RNA-dependent RNA polymerase (RdRp), movement protein (MP), capside protein (CP) and the tRNA-like His structure at the 3' end (3' His). The sequenced region is indicated by dashed lines and is shown enlarged in the lower part. Mutations are represented as black lines on pink background in the RdRp region or purple background in the MP region.

**Table S6. Run test for randomness of mutations found in the RdRp and MP regions at 10 dpi.**

| | 5 dpi | | 10 dpi | | 31 dpi | |
|---|---|---|---|---|---|---|
| | **RdRp** | **MP** | **RdRp** | **MP** | **RdRp** | **MP** |
| **A/U**[a] | 0.969[b] | 0.980 | 0.969 | 0.980 | 0.969 | 0.980 |
| **0**[c] | 0.014 | 0.690 | 0.002 | 1.000 | 0.642 | 0.561 |
| **100**[c] | 0.535 | 0.001 | 0.557 | 0.484 | 0.557 | 0.103 |

[a] Distribution of adenine and uracil along the sequence of the RdRp and MP regions.
[b] The distribution is random for P>0.05
[c] Mutations of untreated (0 µg/ml 5-FU) and treated (100 µg/ml 5-FU, replicates 1, 2 and 3) TMV quasispecies are described in Supplementary Table S2.

**Table S7. Primers used in this study.**

| Primers | Position | Secuence |
|---|---|---|
| **A-4364_F** | 4364-4384 (TMV) | 5’-CGCAGGTATCAAAACTTGCAT |
| **A-5684_R** | 5684-5665 (TMV) | 5´-GTAGCCTCCGAATCATCATC |
| **qTMV1699_R** | 1699-1680 (TMV) | 5'-AGCGCAGGCATGTCCACAGA |
| **qTMVFor1367** | 1367-1387 (TMV) | 5´CGGTGTGACAGCGAGGTCCG |
| **25S_Universal-F** | *Nicotiana* | 5'-ATAACCGCATCAGGTCTCCA |
| **25S_Universal-R** | *bentamiana* | 5'-CCGAAGTTACGGATCCATTT |
| **1225_F** | 1225-1245 (TMV) | 5’-AGAGGACGCGCAAGGAAGTCT |
| **T7** | | 5’-TAATACGACTCACTATAGGG |
| **SP6** | | 5’-ATTTAGGTGACACTATAG |
